# Overexpression of microRNA-99a Attenuates Cardiac Hypertrophy

**DOI:** 10.1371/journal.pone.0148480

**Published:** 2016-02-25

**Authors:** Qiaoling Li, Jun Xie, Bingjian Wang, Ran Li, Jian Bai, Liang Ding, Rong Gu, Lian Wang, Biao Xu

**Affiliations:** 1 Department of Cardiology, Nanjing Drum Tower Hospital, The Affiliated Hospital of Nanjing University Medical School, Nanjing, Jiangsu, 210008, China; 2 From the Department of Cardiology, Drum Tower Clinic Hospital, Nanjing Medical University, Nanjing, China; University of Hawai'i Manoa, UNITED STATES

## Abstract

Pathological cardiomyocyte hypertrophy is associated with significantly increased risk of heart failure, one of the leading medical causes of mortality worldwide. MicroRNAs are known to be involved in pathological cardiac remodeling. However, whether miR-99a participates in the signaling cascade leading to cardiac hypertrophy is unknown. To evaluate the role of miR-99a in cardiac hypertrophy, we assessed the expression of miR-99a in hypertrophic cardiomyocytes induced by isoprenaline (ISO)/angiotensin-II (Ang II) and in mice model of cardiac hypertrophy induced by transverse aortic constriction (TAC). Expression of miR-99a was evaluated in these hypertrophic cells and hearts. We also found that miR-99a expression was highly correlated with cardiac function of mice with heart failure (8 weeks after TAC surgery). Overexpression of miR-99a attenuated cardiac hypertrophy in TAC mice and cellular hypertrophy in stimuli treated cardiomyocytes through down-regulation of expression of mammalian target of rapamycin (mTOR). These results indicate that miR-99a negatively regulates physiological hypertrophy through mTOR signaling pathway, which may provide a new therapeutic approach for pressure-overload heart failure.

## Introduction

Pathological hypertrophy is one of the leading pathological causes of heart failure and sudden cardiac death worldwide. Myocardial stress such as injury, prolonged hypertension or valvular heart disease induces pathological cardiac hypertrophy. Initially, cardiac hypertrophy is believed to be a compensatory response to increased wall tension and/or decreased cardiac output. However, prolonged cardiac hypertrophy progresses to contractile dysfunction, cardiac decompensation, eventually heart failure and sudden cardiac death [1–2]. During the development of cardiac hypertrophy, hemodynamic stress or neuroendocrine signaling evokes physiologic or pathologic remodeling responses through the activation of intra-cellular signaling pathways and transcriptional mediators in cardiomyocytes, leading to increased protein synthesis and cardiomyocytes size [3–4], re-expression of fetal cardiac genes, and assembly of sarcomeres [5]. One of the major signaling pathways regulating cardiac hypertrophy is the pro-hypertrophic mTOR/P70/S6K signaling pathway. Inhibitors of mTOR protect heart from ischemia and overload [6–7].

MicroRNAs are involved in a variety of basic biological processes in cardiovascular system, for example, cardiac fibrosis and apoptosis [8–9], heart stress responses [10–11] and hypertrophy [12]. Targets of miR-99a include a variety of signaling molecules, such as mTOR, SWI/SNF-related matrix-associated actin-dependent regulator of chromatin subfamily A member 5 (SMARCA5), SWI/SNF-related matrix-associated actin-dependent regulator of chromatin subfamily D member 1 (SMARCD1) and fibroblast growth factor receptor 3 (FGFR3) [13–16]. Although miR-99a has been reported to be associated with myocytes proliferation and apoptosis [17–18], to date, study on the precise role and therapeutic potential of miR-99a in cardiac hypertrophy is still lacking. Here, we investigated the functional role of miR-99a in cardiac hypertrophy using a TAC mice model and ISO/Ang II stimulated cardiomyocytes. We observed that miR-99a overexpression protected cardiomyocytes from ISO/Ang II induced hypertrophy. Cardiac-specific overexpression of miR-99a protected hearts from TAC induced cardiac hypertrophy and subsequent heart failure, and attenuated the re-activation of fetal cardiac genes in the adult failing heart. Taken together, our research showed a previously unknown link between miR-99a and cardiac hypertrophy, and provide a promising therapeutic strategy for pathological cardiac hypertrophy.

## Material and Methods

### Animal Care and Use

The investigation conforms to the Guide for the Care and Use of Laboratory Animals published by the US National Institutes of Health (NIH Publication No. 85–23, revised 1996) and was approved by the Ethics Review Board for Animal Studies of Nanjing Drum Tower Hospital.

### Primary Culture of Neonatal Mice Ventricular Myocytes (NMVMs)

Newborn CD1 mice (1–2 day old) were sacrificed by decapitation. Hearts were quickly excised, and the atria were removed and the ventricles were transferred into ice-cold Hank’s balanced salt solution (HBSS) without Ca^2+^ and Mg^2+^ (Gibco, Invitrogen, Carlsbad, CA), then rapidly minced in Trypsin-EDTA 0.125% (Invitrogen) [19] at 4°C overnight (within 24 hours). Collagenase (Invitrogen, 0.5 mg/ml in DMEM) was used to further digest the tissues in shaking bath at 37°C within 10 minimums. NMVMs were collected 1.5 hours later from the supernatants and cultured with DMEM containing 1g/L glucose plus 10% FBS and 1% penicillin/streptomycin (GIBCO). The protocol was approved by the Institutional Animal Care and Use Committee of the affiliated Drum Tower Hospital of Nanjing University Medical School.

### Recombinant Lentivirus Construction

MiR-99a sequence forward 5'- GAAGACCTTTACTGGGAATA-3', reverse 5'-GCCTTGAATGGCTCTGCTAC-3'. Negative control: TTCTCCGAACGTGTCACGT (Genepharma, Shanghai). MiR-99a sequence was inserted into Pglv3/h1/gfp+puro plasmid vector (Genepharma, Shanghai) and driven by H1 promoter. The pseudoviral particles were produced using lentivector packaging system (Genepharma, Shanghai) according to the manufacturer's instructions. Lentivirus carried mir-99a inhibitor (LV-anti-GFP) sequence (5’-3’CACAAGATCGGATCTACGGGTT) were purchased from Shanghai GenePharma. NMVMs were infected at a multiplicity of infection (MOI) of 50.

### Mice TAC model

C57BL/6 male mice (8 weeks old, 18–23 g) were divided into 2 groups: 1). Group #1 mice received thoracotomy without (shame, n = 6) or with TAC. Heart samples were collected to evaluated the expression of miR-99a and mTOR 1 week (n = 11) and 8 weeks (n = 16) after TAC; 2). Group #2 mice were subject to chest reopening without (sham, n = 6) or with injection of 35ul lentivirus (3.5*10^7^ viral particles per mice) containing either miR-scramble GFP (Lenti-GFP group, n = 25) or miR-99a precursor GFP (Lenti-99a-GFP group, n = 10) in the heart 1 week after TAC operation. 35ul lentivirus containing either miR-99a precursor GFP or miR-scramble GFP (3.5*10^7^ viral particles).

Mice were anesthetized by intraperitoneal administration of a mixture of ketamine hydrochloride (50 mg/kg) and diazepam (2.5 mg/kg). Absence of response to painful stimuli was used as indicative of adequate anesthesia. Mice were endotracheally intubated and mechanically ventilated (Jiangxi Teli, China) with supplemental oxygen. The TAC operation was performed as described [20].

### UCG and Hemodynamic Assessment

The M-mode measurements of left ventricular (LV) dimensions were averaged from more than 3 cycles. LV end-systolic diameter (LVID;d) and end-diastolic diameter (LVID;s) were measured. Percent LV fractional shortening (FS%) was calculated as follows: FS% = (LVID;d—LVID;s)/LVID;d*100(%). Percent ejection fraction (EF%): EF% = 100*((LV Vol;d—LV Vol;s)/LV Vol;d). LV Vol;d = ((7.0/(2.4+LVID;d)) * LVID;d^3^); LV Vol;s = ((7.0/(2.4+LVID;s)) * LVID;s^3^).

For hemodynamic assessment, mice were maintained in a 37°C metal chamber. Systolic blood pressure (SBP), diastolic blood pressure (DBP) and heart rate were measured using a programmable tail-cuff sphygmomanometer (BP-98A; Softron, Tokyo, Japan). Training measurements were made for 3 days to acclimatize the animals to the machine, followed by 2 days of recorded measurements. Three sets of 5 measurements were taken daily for each mouse, and the 1st set was discarded. To eliminate bias caused by struggling or other physiological alterations, each set of measurements was accepted only if the standard deviation of the set was < 9 mmHg.

### Histological Examination

Hearts from mice at 1 week and 8 weeks after TAC were fixed with formalin, embedded with paraffin and cut into 4 μm slices for histological analysis. The size of heart was assessed by histological analysis with hematoxylin / eosin (HE) staining and Masson staining.

Paraffin-embedded tissue sections were stained with mouse monoclonal α-sarcomeric actin (α-actin) antibody (1:75, Abcam) and Alexa Fluor 594 goat anti-mouse antibody (1:200, Molecular Probes) as secondary antibody. DAPI (4’-6-diamidino- 2-phenylindole, Sigma) were used as nuclei marker. Sections were mounted and analyzed with a fluoview 1000 confocal microscope (Olympus, Japan).

### RNA Isolation and Reverse Transcription

Total RNA was extracted using TRIzol (Invitrogen) according to the manufacturer’s instructions. For microRNA RT—PCR, TaqMan microRNA assays (Applied Biosystems) were used to quantify the expression of mature miR-99a (Assay ID 000435) and U6 (Assay ID: 001973). MiR-99a expression was relative to the control U6. Amplication and detection were performed using 7500HT Fast Real-Time PCR system (Applied Biosystems). Relative expression was calculated using the comparative Ct method (2-^[△][△]Ct^) [21].

The sequences of primers used for PCR are listed in S1 Table. The reactions contained 2 × SYBR Premix Ex TaqTM (Takara), 50 × Rox Reference Dye II, each primer at 200 nM, and 2μL of cDNA template in a 20μL reaction volume. Amplification was performed with an initial denaturation step at 95°C for 30 s, followed by 40 cycles of denaturation at 95°C for 5 s, annealing and extension at 60°C for 34 s. After amplification, the amplification specificity was confirmed by melting-curve analysis of the PCR products. The qRT-PCR was performed using the ABI Prism 7500 device. All samples were run as triplicates. β-actin was used as the house keeping gene.

### Western Blotting Analysis

Adult male C57/BL6 mice were sacrificed at 8 weeks after TAC or sham surgery and heart samples were collected. Proteins were then extracted from these LV of hearts and assessed by western blotting analysis. The primary antibodies used were antibodies against P70/S6K, FGFR3, GAPDH (Bioworld Technology, Inc.), phosphor-P70/S6K and Caspase 3 (Cell Signaling Technology, Inc.), mTOR (Abcam, Inc), α-smooth muscle actin (α-SMA) (Abcam, Inc), atrial natriuretic peptide (ANP) (Abcam, Inc), SMARCD1 and SMARCA5 (ProteintechGroup, Inc.).

### TUNEL Assay

Cellular apoptosis was evaluated using DeadEnd Fluorimetric TUNEL System (Roche) according to the manufacturer’s instructions. Paraffin-embedded tissue sections were counterstained with mouse monoclonal α-sarcomeric actin antibody (1:75, Abcam) and Alexa Fluor 633 goat anti-mouse antibody (1:250, Molecular Probes) as secondary antibody. DAPI as nuclei marker. Sections were mounted and analyzed using a Fluoview 1000 confocal microscope (Olympus, Japan). The number of TUNEL-positive cardiomyocytes nuclei was manually determined. The total number of nuclei (exhibited as DAPI-positive signals) was automatically calculated using Image Pro Plus software (Media Cybernetic).

### Statistical Analysis

Data were expressed as mean ± SEM. All data analysis was performed using SPSS 13.0 software (SPSS, Inc, Chicago, IL, USA). Statistical significance was defined as P<0.05 (two-tailed). The normality or otherwise of distribution of the continuous variables was assessed with the Shapiro-Wilk test. Comparison of parameters between two groups was performed by unpaired Student's t test (where distributions were normal) or Mann-Whitney U test (where distributions were significantly skewed). The authors had full assess to, and take full responsibility for, the integrity of the data. All authors have read and agreed to the manuscript as submitted. When multiple comparisons were made, statistical significance was determined using one-way ANOVA followed by Tukey’s post-test.

## Results

### Expression of mTOR and MiR-99a in Hypertrophic and Failing Mouse Hearts

**Fig 1:**
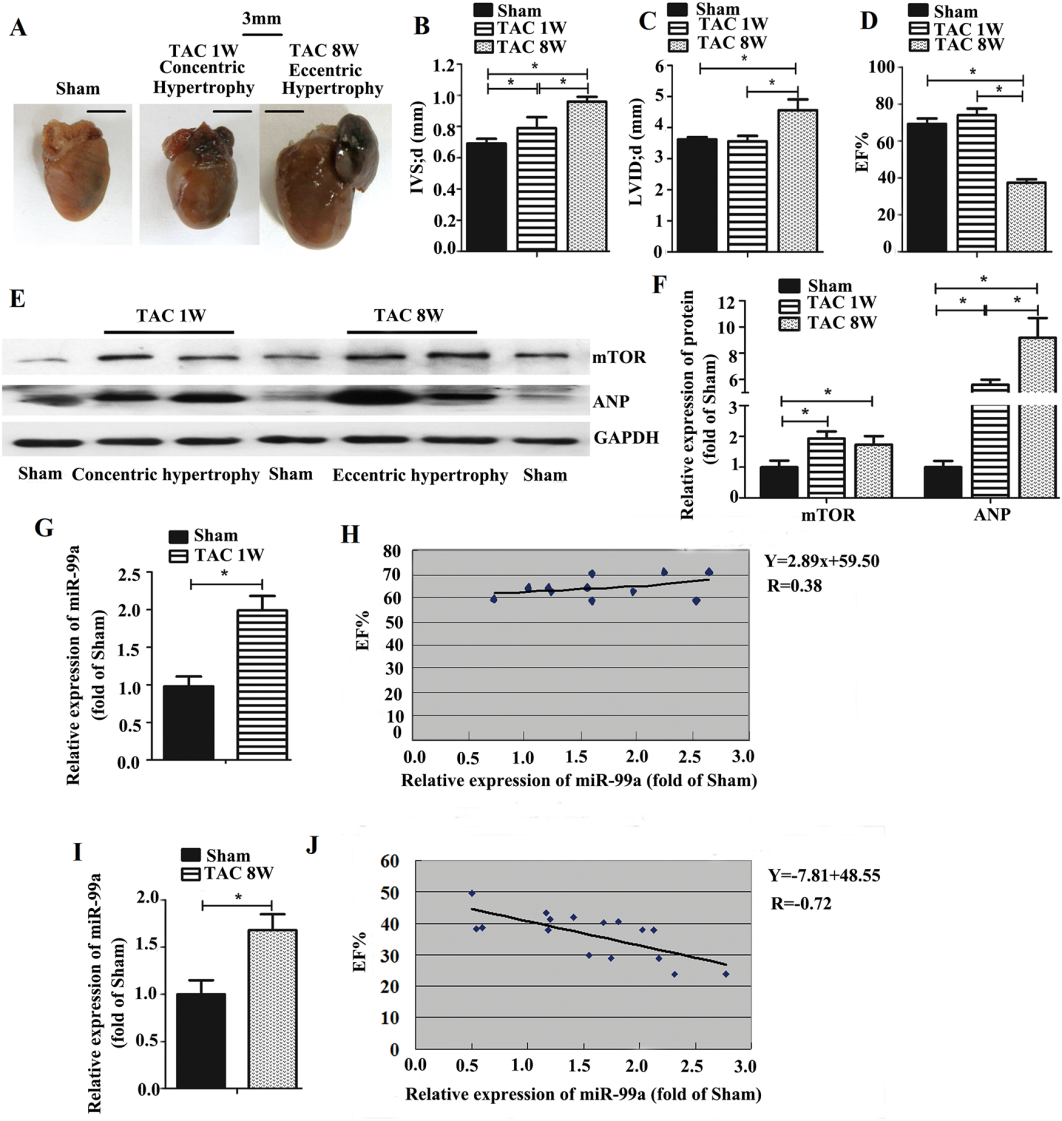
The expression of miR-99a and mTOR in heart with hypertrophy and failure induced by TAC in mice. A. Representative images of hearts. B-D. UCG measurement after TAC surgery. There was no significant difference in EF% and LVID;d between sham and TAC group 1 week after surgery (concentric hypertrophy). Decreased EF% and enlarged LVID;d were observed in TAC group 8 weeks after surgery (eccentric hypertrophy). IVS;d was increased both 1 week and 8 weeks after surgery. E-F. mTOR expression was increased 1 week and 8 weeks after TAC. G-H. MiR-99a expression was increased 1 week after surgery, but low correlated with cardiac function. I-J. MiR-99a expression was increased 8 weeks after surgery, and showed a high correlated with cardiac function. (1 week after TAC: n = 11; 8 weeks after TAC: n = 16; Sham group: n = 6). *, p<0.05.

UCG analysis shows no significant cardiac enlargement in mice 1 week after the introduction of TAC, and cardiac enlargement became prominent 7 weeks later (Fig 1A). One week after TAC, the hearts were able to produce a compensatory response (concentric hypertrophy) to left ventricular pressure overload, as demonstrated by increased wall thickness (Fig 1B) and EF% (Fig 1D). However, eight weeks after TAC, the heart progressed to decompensation with sustaining exposure to pressure overload. It leads to eccentric cardiac hypertrophy accompanied by addition of new sarcomeres in series with existing sarcomeres, as demonstrated by increased IVSd and LVIDd, decreased EF% (Fig 1B–1D), and up-regulated ANP (Fig 1E and 1F) 8 weeks after TAC. These findings indicated that the hearts developed compensatory hypertrophy 1 week after TAC surgery, and deteriorated into failure 8 weeks after TAC surgery.

To investigate whether mTOR is involved in the development of cardiac hypertrophy, we assessed mTOR protein expression using western blotting analysis. As shown in Fig 1, mTOR expression was increased in the hearts 1 week (concentric hypertrophy) and 8 weeks (eccentric hypertrophy) after TAC (Fig 1E and 1F). Expression of miR-99a during the development of concentric hypertrophy and eccentric hypertrophy was assessed by Taqman RT-PCR analysis. One week after surgery, hearts in TAC group developed concentric hypertrophy, and miR-99a level was about 2-fold higher in those group than the sham group (Fig 1I); whereas, eight weeks after TAC, hearts developed eccentric hypertrophy and the miR-99a level was about 1.74 fold higher in TAC group (Fig 1K). To investigate whether there is a connection between miR-99a level and LV EF%, we performed correlation analysis between these two sets of data. The expression of miR-99a showed low correlated with cardiac function (R = 0.38, p = 0.22) 1 week after surgery (Fig 1J), while miR-99a expression showed a high inverse correlation with cardiac function (R = -0.72, p<0.01) eight weeks after surgery (Fig 1L), suggesting that miR-99a might correlate with hypertrophic growth of cardiomyocytes and affect the heart function in vivo.

### Expression of mTOR and MiR-99a in NMVMs upon Hypertrophic Stimuli Treatment

**Fig 2:**
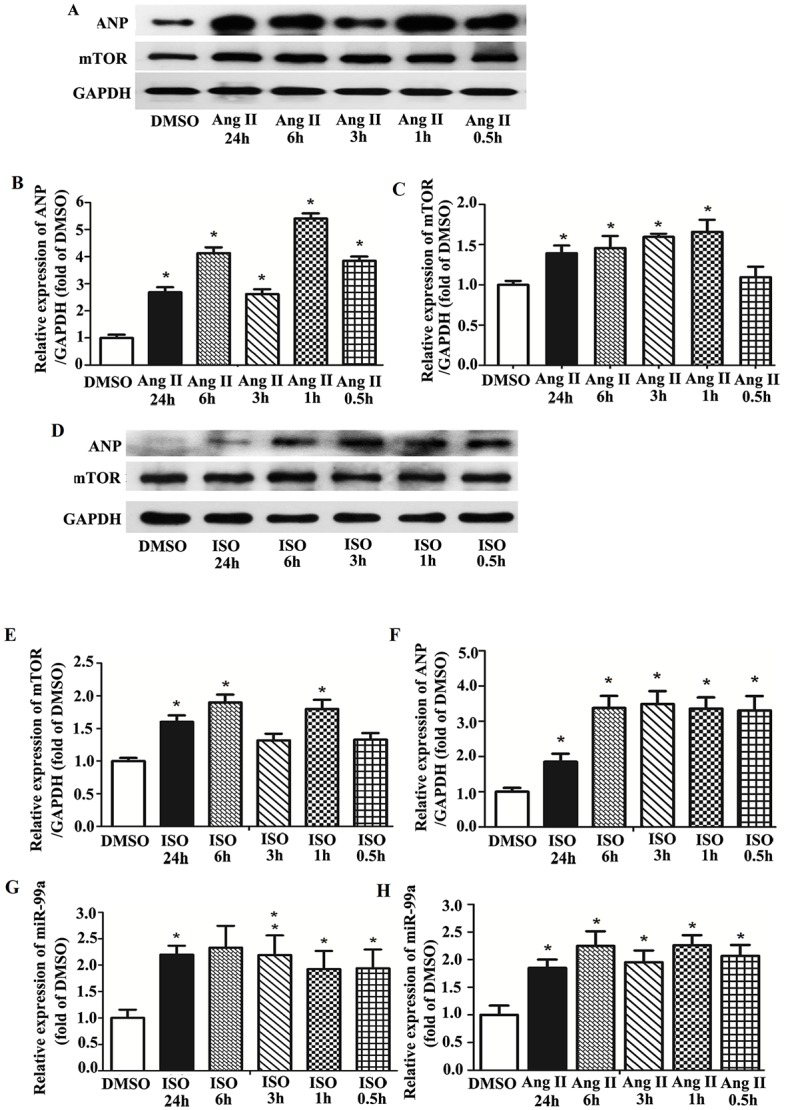
The expression of miR-99a and mTOR in NMVMs upon hypertrophic stimuli. A. Expression of mTOR and ANP in NMVMs under Ang II stimulation assessed by western blotting analysis. B. ANP expression was up-regulated at least 2.68-fold upon Ang II stimulation. C. Increased mTOR expression was observed as early as 1 hour after Ang II stimulation (increased about 1.60-fold), and lasted for at least 24 hours. D. Expression of mTOR and ANP in NMVMs under ISO stimulation assessed by western blotting analysis. E. mTOR expression was increased under ISO stimulation. F. ANP expression was up-regulated upon ISO stimulation. G. MiR-99a expression was increased about 2.00-fold after ISO stimulation. H. MiR-99a was increased at least 2.00-fold upon Ang II stimulation. Each experiment was repeated more than 3 times. *, p<0.05.

To investigate whether miR-99a/mTOR signaling pathway is involved in cardiomyocytes hypertrophy, we assessed the expression of miR-99a and its target mTOR in hypertrophic NMVMs. The purity of α-actin^+^ cell was ≥95% in culture cell population (S1A Fig), suggesting that most cells are NMVMs. The expression of ANP, a biomarker of cardiomyocyte stress, was detected to prove the successful induction of hypertrophic cardiomyocytes model using western blotting. The ANP expression and mTOR expression were up-regulated in both Ang II (10 μmol/L) (Fig 2A–2C) and ISO (20 μmol/L) (Fig 2D–2F) treated NMVMs at different time points.

Taqman RT-PCR was used to determine the changes of miR-99a expression in NMVMs under stimuli. Expression of miR-99a was up-regulated in NMVMs under Ang II (Fig 2G) or ISO (Fig 2H) stimulation, suggesting that miR-99a was functionally involved in the development of hypertrophy in cardiomyocytes induced by different stimuli *in vitro*.

Taken together, these results demonstrated that both mTOR and miR-99a expressions were increased in cardiomyocytes during cardiomyocyte hypertrophy in vitro.

### MiR-99a Inhibits Cardiomyocytes Hypertrophy in vitro

**Fig 3:**
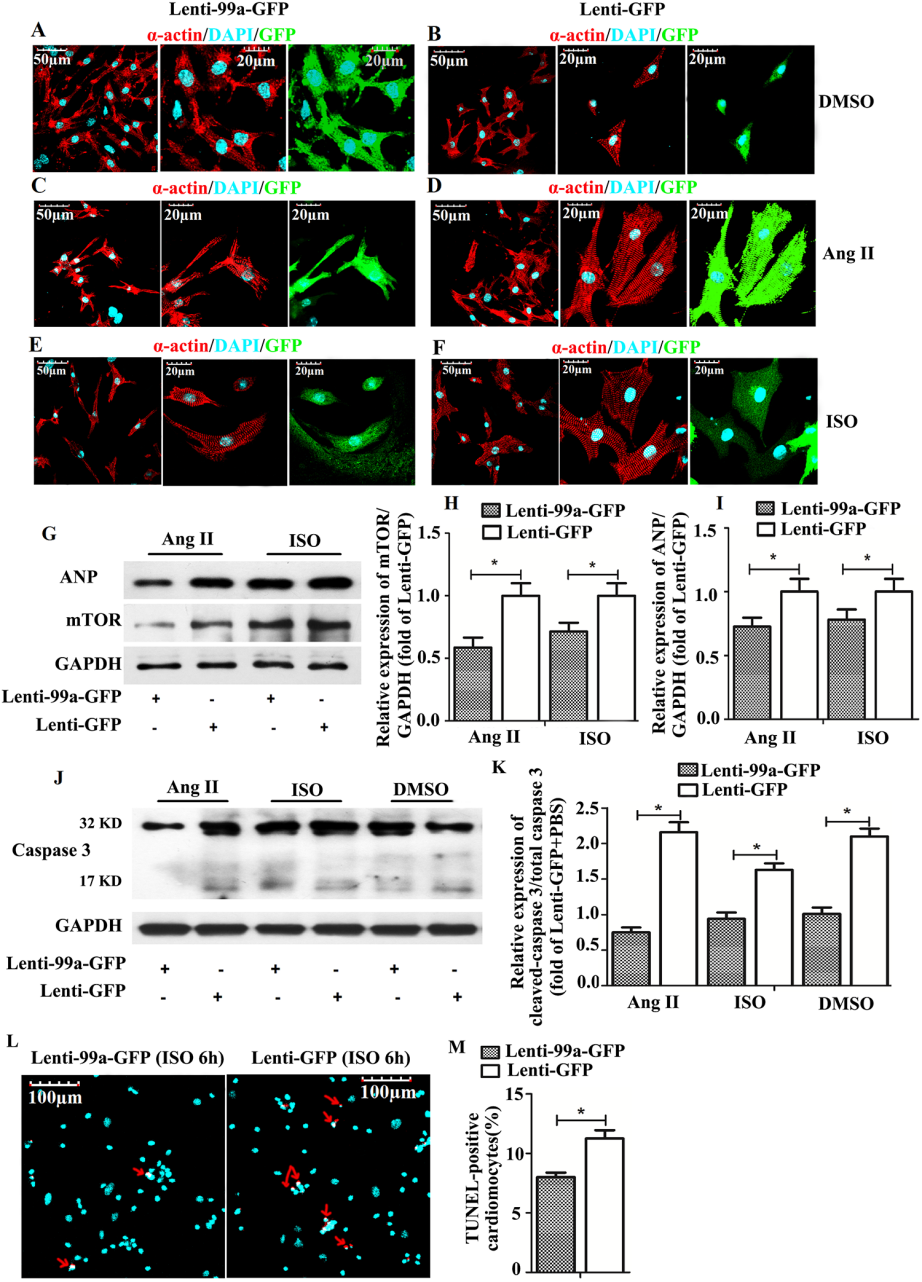
The anti-hypertrophic role of MiR-99a in NMVMs under hypertrophic stimuli. A-B. NMVMs transfected with lenti-GFP and lenti-99a-GFP without ISO or Ang II stimulation. C-F. NMVMs transfected with lenti-GFP or lenti-99a-GFP were treated with 10 μmol/L Ang II (C, D) for 6 hours or 20 μmol/L ISO (E, F) for 6 hours. G-I. Inhibition of mTOR and ANP expression by miR-99a in NMVMs under ISO or Ang II stimulation assessed by western blotting analysis. J-M. Decreased cell apoptosis in MiR-99a overexpressing NMVMs under ISO or Ang II stimulation assessed by western blotting analysis and TUNEL assay. *, p<0.05.

To identify whether miR-99a overexpression protects cardiomyocytes from stumili-induced hypertrophy, we transfected NMVMs with lentivirus vector containing miR-99a. Seventy-two hours after transfection, miR-99a overexpressing NMVMs (about 22-fold increase) were exposed to hypertrophic stimuli. The average NMVMs cell size was increased in NMVMs without miR-99a lentiviral transfection in the presence of Ang II (Fig 3D) or ISO (Fig 3F), as compared to miR-99a overexpressing NMVMs (Fig 3C–3E), suggesting that miR-99a overexpression restored cardiomyocyte phenotype under hypertrophic stimuli. To investigate whether mTOR, the downstream molecule of miR-99a, is involved in this process, we assessed mTOR protein expression in miR-99a overexpressing cells. Western blotting analysis showed an up to 40% decrease in mTOR protein level in NMVMs after miR-99a lentiviral infection (date not shown). Overexpression of miR-99a reduced mTOR expression in Ang II-induced hypertrophic NMVMs (Fig 3G). ANP expression was decreased in miR-99a treated cells under Ang II stimulation compared to lenti-GFP group (Fig 3G–3I), suggesting that miR-99a overexpression protects cells from hypertrophic stress. We observed the similar result in ISO treated group (Fig 3G and 3H).

We further evaluated the protective role of miR-99a in hypertrophic stress by assessing cell apoptosis upon stimuli. Western blotting analysis of cleaved caspase-3 and TUNEL assay revealed decreased apoptosis in lenti-99a-GFP group in the presence of ISO (Fig 3J–3M) or Ang II stimuli (Fig 3J and 3K). The cardiomyocytes were infected with LV-anti-GFP or lentivirus contained negative sequence (Lenti-GFP) for 48 or 72 hours. We observed the expression of mir-99a was declined to 24% of its original value in LV-anti-GFP group (date not show). We observed that inhibition of miR-99a could do harm to cardiomyocytes under the treatment of Ang II and ISO. Cardiomyocytes were infected with lentivirus for 72 hours, then treated with Ang II (1/10umol/L) or ISO (1/10umo/L) for 12 hours. We found that cardiomyocytes suffered from Ang II (1umol/L and 10 umol/L) and ISO (10umol/L) showed more TUNEL-positive cells in LV-anti-GFP group than lenti-GFP group (S2 Fig). There was no obvious difference of TUNEL-positive cells under ISO (1umol/L) stimulating between LV-anti-GFP and lenti-GFP group.

Taken together, these results demonstrated that miR-99a/mTOR signaling pathway inhibits hypertrophic growth of cardiomyocytes under pathological stimuli *in vitro*, and protects cardiomyocytes from apoptosis under hypertrophic stimuli.

### MiR-99a Overexpression Inhibits Cardiac Hypertrophy *in vivo*

**Fig 4:**
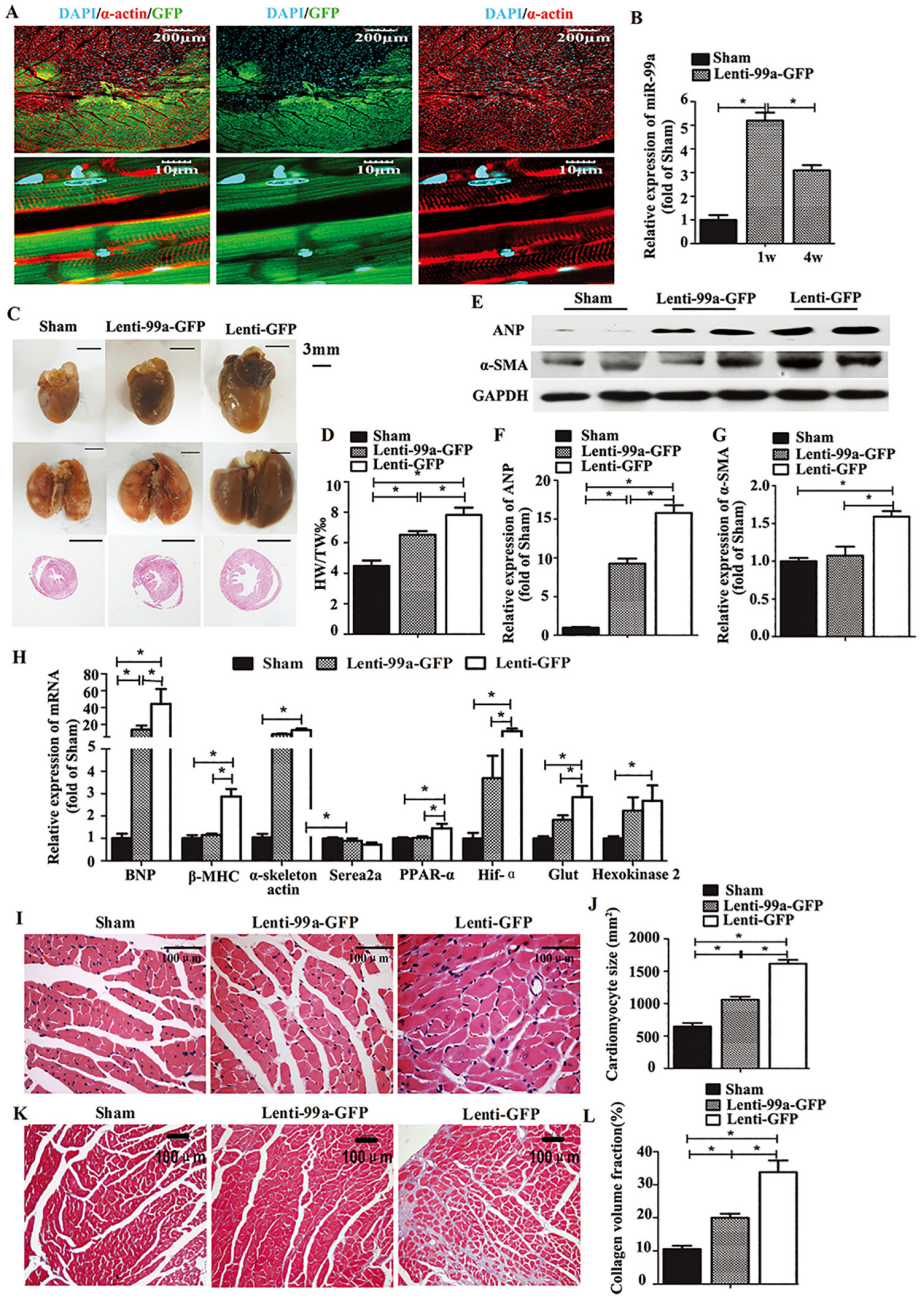
MiR-99a gene therapy attenuates cardiac hypertrophy in a mice model with TAC. A, Immunofluorescence for GFP tag, indicating successful expression of exogenous miR-99a in heart after lenti-99a-GFP delivery. (α-actin: red; GFP: green; DAPI: cyan). B. Increased miR-99a expression in heart 1 week and 7 weeks after lentiviral delivery assessed by TaqMan RT-PCR. C More spherical shape of hearts from lenti-GFP mice (right column) comparing to lenti-99a-GFP (middle column) and sham mice (left column). Bigger size of lungs from lenti-GFP mice (right column) relative to lenti-99a-GFP (middle column) and sham mice (left column). The bottom panel shows paraffin-embedded sections of the hearts from the top panel. Bars = 3mm. D. Heart-to-body-weight ratio (mg/g) was increased in TAC mice, but attenuated in miR-99a overexpressing heart (n = 8 in lenti-99a-GFP group; n = 19 in lenti-GFP group). E-G. Western blotting analysis showed ANP (F) and α-SMA (G) were both down-regulated in miR-99a treated group compared to lenti-GFP group. H. BNP, β-MHC, PPAR-α, ACTA1, Hif-α, GLUT1 and HK2 levels were strongly increased in lenti-GFP treated hearts, but attenuated in lenti-99a treated hearts. Serca2a was decreased after surgery, but there was no significant difference in Serca2a expression among these three groups. I-J. Analysis of cardiomyocytes size in HE—stained sections (n = 3–5 per each group). K-L. Analysis of cardiac fibrosis in Masson—stained sections (n = 3–5 per each group). *, p<0.05.

To elucidate whether miR-99a overexpression was sufficient to decrease cardiac hypertrophy in vivo, we generated mice with cardiac specific overexpression of miR-99a through lentivirus infection 1 week after the TAC surgery. We observed the expression of GFP on heart slices from the mice 1 week (Fig 4A) and 7 weeks (date not shown) after lentivirus delivery. Overexpression of miR-99a in heart tissues from the mice 1 week and 7 weeks after lentiviral delivery was verified by Taqman RT—PCR analyses of the mature forms of these miRNAs (Fig 4B). No significant change of miR-99a expression was seen in kidney, lung, liver and spleen, indicating the successful lentiviral transfection in heart (S1B Fig). TAC induced pathological hypertrophy in mice as reflected by increased heart weight indices and the overall heart size, and these TAC-induced changes were attenuated in miR-99a treated mice (Fig 4C and 4D).

The expression of cardiac stress marker ANP was dramatically decreased in lenti-99a-GFP hearts compared to lenti-GFP hearts (Fig 4E and 4F), demonstrating that miR-99a overexpression abates the development of heart failure in TAC mice. Reactivation of the fetal cardiac gene program in adult hearts is a reliable marker of cardiac hypertrophy and heart failure [22]. In our study, the ‘fetal’ genes including BNP, β-MHC and ACTA1 levels were strongly increased in lenti-GFP treated hearts (Fig 4H), and reduced in lenti-99a-GFP group, further supporting our observation that miR-99a overexpression attenuated cardiac hypertrophy and heart failure. It has been reported that the cardiac isoform of the sarcoplasmic reticulum calcium ATPase pump (SERCA2a) expression is positively correlated with heart function [23]. We observed restored serca2a expression in miR-99a treated heart compared to lenti-GFP group, indicating improved heart function with miR-99a treatment.

Expressions of genes associated with metabolism are changed due to the altered glucose and lipid metabolism in hypertrophic heart, for example, Hif-1α, PPAR-α, GLUT1 and HK2 expressions are increased in this situation [24]. To investigate whether miR-99a overexpression affects cardiomyocytes metabolism, we analyzed Hif-1α, PPAR-α, GLUT1 and HK2 mRNA expression in hearts by RT-PCR. All of the four genes were up-regulated in TAC group compared to sham group, and were significantly decreased or tend to decrease in miR-99a treated group (Fig 4H), suggesting improved cardiac metabolism in miR-99a treated heart.

**Fig 5:**
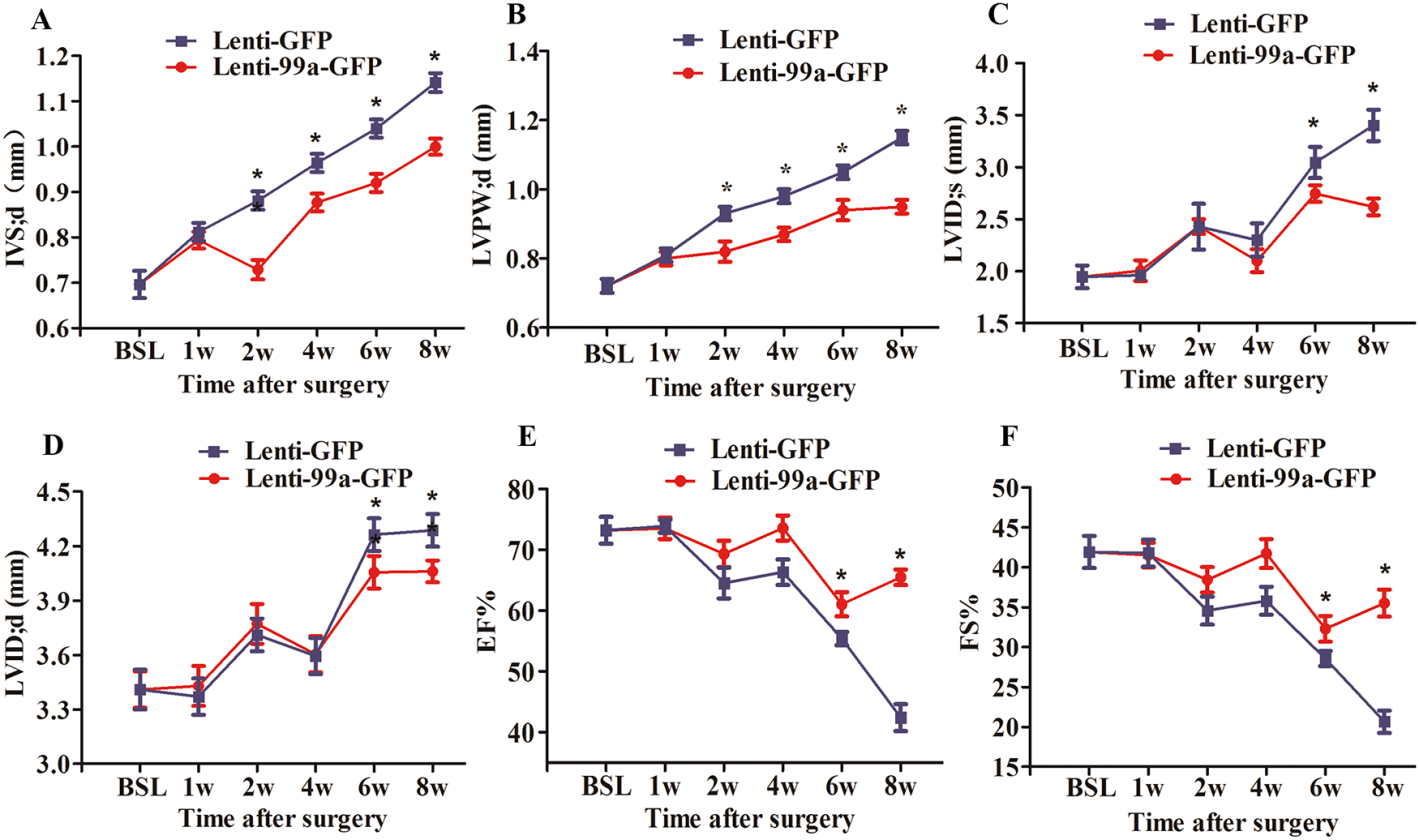
MiR-99a suppressed hypertrophy and improved cardiac function in response to pressure overload. A. Interventricular septum thickness was decreased as early as 1 week after MiR-99a lentiviral delivery, and this effect lasted for at least 7 weeks. B. Hypertrophy of LV posterior wall was attenuated in TAC mice with treatment of miR-99a as early as 1 week after lentiviral delivery. C-D. LVID;s (C) and LVID;d (D) were enlarged in response to pressure overload. MiR-99a treated group showed preserved LVID;s and LVID;d at 5 weeks and 7 weeks after lentiviral delivery. E-F. EF% and FS% were improved in TAC mice with miR-99a treatment at 5 weeks and 7 weeks after infection. IVS, interventricular septum; LVID;d, LV end-diastolic diameter; LVID;s, LV end-systolic diameter; LVPW, LV posterior wall; %EF, Ejection fraction; %FS, percent fractional shortening. *, p<0.05.

Cardiomyocytes size as determined in histological sections of hearts was significantly decreased in miR-99a treated group compared to lenti-GFP group (Fig 4I and 4J). TAC-induced cardiac fibrosis, a hallmark of cardiac remodeling, was less profound in miR-99a treated hearts (Fig 4K and 4L). Moreover, western blotting analysis showed decreased expression of a myofibroblast marker α-SMA [25] in Lenti-99a-GFP group after TAC surgery (Fig 4E and 4G). These data demonstrated that miR-99a overexpression improves heart morphology after TAC. We also evaluated mice cardiac function using small animal UCG. Interventricular septum was significantly decreased in in miR-99a group as early as 1 week after lentiviral delivery, and lasted for 7 weeks (Fig 5A). LVPW;d, LVID;s and LVID;d were significantly reduced in miR-99a treated heart compared to the control ones at different time points (Fig 5B–5D). EF% (Fig 5E) and FS% (Fig 5F) were significantly improved in lenti-99-GFP group at 5 weeks, and 7 weeks after lentiviral delivery.

To investigate whether miR-99a overexpression also improves hemodynamics in mice, we assessed systemic blood pressure (BP) of mice 8 weeks after TAC surgery. Cardiac overexpression of miR-99a improved BP in mice received TAC surgery (systolic BP: 109 ± 3 mmHg and 95 ± 2 mmHg, diastolic BP: 80 ± 2 mmHg and 66 ± 2 mmHg, lenti-99a-GFP group versus lenti-GFP group, *, p<0.05), while systemic blood pressure was comparable between sham and lenti-99a-GFP group (S1C Fig).

Taken together, these results demonstrated that the cardiomyocytes specific overexpression of miR-99a in mice protects heart from pathological hypertrophy and failure.

### MiR-99a Inhibits Cardiac Hypertrophy via an mTOR/P70/S6K Signaling Pathway

**Fig 6:**
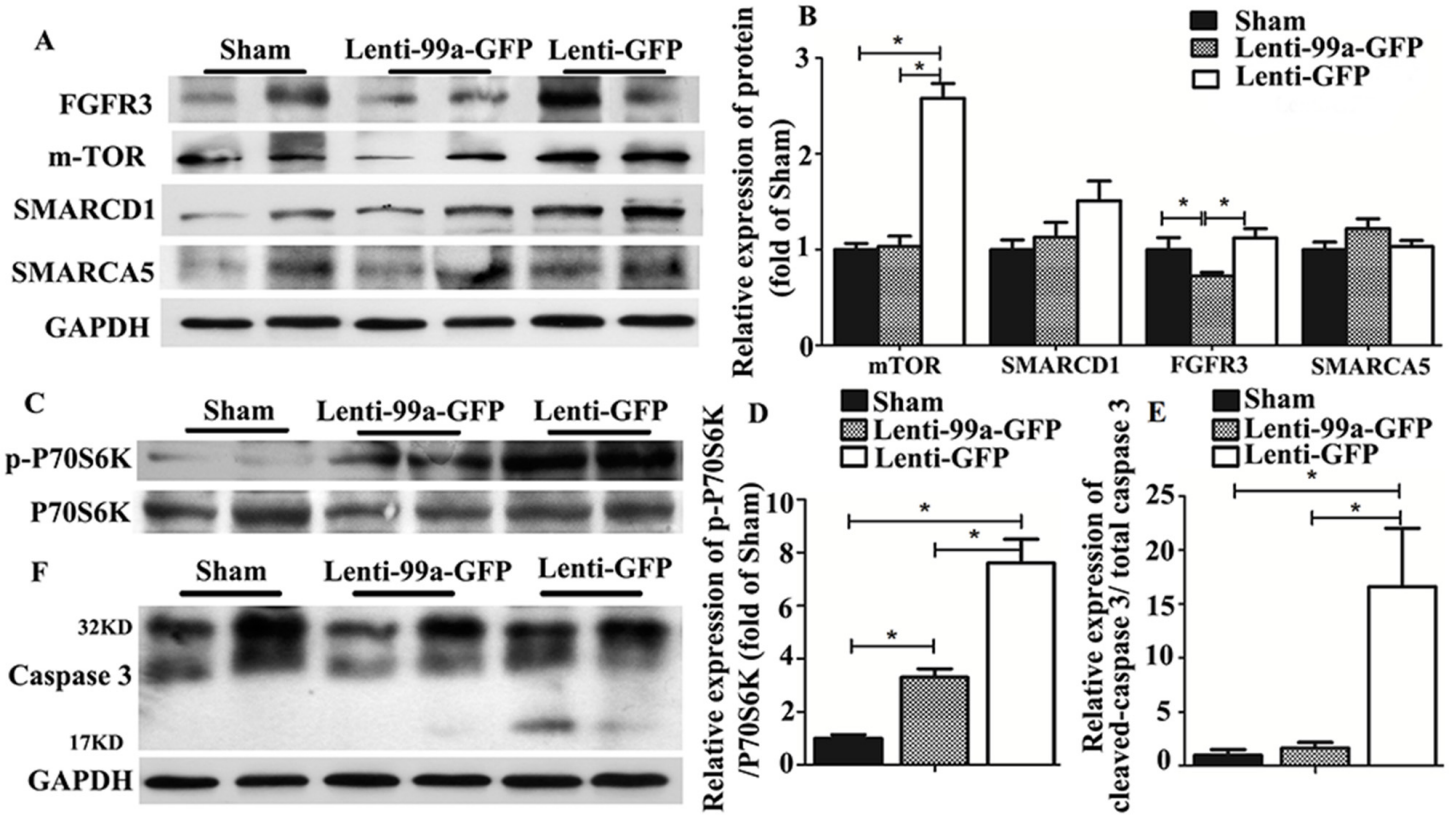
MiR-99a regulated mTOR/P70/S6K signaling pathway and decreased cell apoptosis in heart of TAC mice. A-B. The expression of mTOR, SMARCA5, SMARCD1 and FGFR3 in heart of TAC mice. C-D. Phosphorylation of p70/S6-kinase was significantly decreased in lenti-99a-GFP group compared to lenti-GFP group. E-F. Decreased cleavage of caspase 3 at 17 kD in lenti-99a-GFP group compared to lenti-GFP group. n = 5 per group; *, p<0.05.

Data exist show that mTOR expression could be regulated by miR-99a, and mTOR/P70/S6K signaling pathway played an important role in cardiac hypertrophy. To investigate whether miR-99a improves heart function after TAC via down-regulation of mTOR/P70/S6K pathway in vivo, we assessed mTOR and P70/S6K expression in lentiviral infected heart by western blotting analysis. Both mTOR expression (Fig 6A and 6B) and P70/S6K activation (Fig 6C and 6D) were decreased 7 weeks after miR-99a lentiviral delivery in TAC animals. We observed a 50% decrease in mRNA transcriptional level of mTOR in lenti-99a-GFP group compared to lenti-GFP group (S1D Fig).We also detected the protein expression of other known targets of miR-99a, including SMARCA5 and SMARCD1. No significant decrease expression of these targets were observed in miR-99a treated mice (Fig 6A and 6B), ruling out the involvement of SMARCA5 and SMARCD1 in miR-99a mediated cardiac protection. Intriguingly, we found that another target of miR-99a FGFR3 was down-regulated in lenti-99a-GFP group.

Cell apoptosis plays an important role in hypertrophy related heart failure [26]. To assess whether the attenuation of hypertrophy by miR-99a is due to reduced cell apoptosis in the hearts, we examed cardiomyocytes apoptosis in the hearts of TAC mice. Decreased cleavage of caspase 3 at 17 kD indicates a reduced apoptosis in TAC mice treated miR-99a (Fig 6E and 6F).

Taken together, these results indicated that miR-99a inhibits cardiac hypertrophy via an mTOR/P70/S6K Signaling Pathway.

## Discussion

Here we demonstrated that miR-99a played a key role in cardiac hypertrophy and heart failure for the first time. MiR-99a was up-regulated in hypertrophic stimuli treated cardiomyocytes and pressure-overloaded heart. Overexpression of miR-99a protected cardiomyocytes from stimuli-induced pathological hypertrophy in vitro. Cardiac specific overexpression of miR-99a in pressure-overloaded heart preserved myocardial structure, reduced myocardial fibrosis and apoptosis, attenuated cardiac hypertrophy and improved cardiac function.

Cardiac hypertrophy in response to pathological stresses is an adaptive response to the increase in cardiac load. Initially, the increase in heart mass serves to normalize wall stress, and the heart can function normally at rest. This adaptation is referred to as compensated hypertrophy. However, if the stimulus for pathological hypertrophy is sufficiently intense or prolonged, the ventricle dilates, cardiac function diminishes, and the heart fails (decompensated hypertrophy) [27].

Protein synthesis contributes to the remodeling process of cardiac hypertrophy [28]. As the target of miR-99a, mTOR is known to play a key role in regulating cellular protein synthesis not only in physiological hypertrophy but also pathological remodeling of the heart [29–30]. mTOR inhibitors such as rapamycin has a protective effect on the setting of TAC caused LV hypertrophy via direct inhibition of mTOR in vivo [31–32]. mTOR inhibitors are also shown to inhibit the expression of collagen and α-SMA [33]. A recent study demonstrates that heart failure can be prevented by systemic rapamycin administration before workload (TAC) in vivo [7]. The authors further pointed out that the improvement in cardiac power in rapamycin-treated rats gives credence to the hypothesis that the hypertrophic process may not be necessary to maintain systolic function in hearts subjected to increased workload [7], which is consistent with our results.

We noticed that FGFR3 was also down-regulated in mice heart treated with miR-99a. FGFR3 has been reported to be associated with several conditions, including achondroplasia [34], thanatophoric dwarfism and bladder cancer [35]. However, the role of FGFR3 in cardiac hypertrophy and heart failure is unknown and needs further investigation.

## Conclusion

In all, our results showed that miR-99a negatively regulated mTOR expression and P70/S6K activation. The mTOR/P70/S6K signaling pathway has been shown to play a pro-hypertrophic role in cardiomyocytes under stress. Therefore, miR-99a overexpression may protect heart from hypertrophy via inhibition of mTOR/P70/S6K signaling pathway. Our data suggested that miR-99a overexpression might work as a novel avenue for the treatment of pressure-overload heart disease.

## Supporting Information

S1 Fig A. Purity and lentiviral infection of NMVMs. The purity of α- actin + cells was ≥95% in culture cell population. B. One week after lentivirus (lenti-GFP-99a) intramyocardially infection, there was no difference of miR-99a expression in kidney, lung, liver and spleen. C. We observed that miR-99a overexpression in hearts of mice did alter the BP (systolic BP: 109 ± 3 mmHg and 95 ± 2 mmHg, diastolic BP: 80 ± 2 mmHg and 66 ± 2 mmHg, lenti-99a-GFP group verse lenti-GFP group, *, p<0.05). However, systemic blood pressure of sham group and lenti-99a-GFP group were not different. D. Seven weeks after infection, we observed a 50%-fold decrease in mRNA transcriptional level of mTOR in lenti-99a-GFP group compared to lenti-GFP group (*, p<0.05).
(TIF)

S2 Fig Inhibition of miR-99a could do harm to cardiomyocytes under the treatment of Ang II and ISO. Cardiomyocytes were infected with LV-anti-GFP or Lenti-GFP for 72 hours, and then treated with Ang II (1/10umol/L) or ISO (1/10umo/L) for 12 hours. We found that cardiomyocytes suffered from Ang II (1umol/L and 10 umol/L) and ISO (10umol/L) showed more TUNEL-positive cells in LV-anti-GFP group than lenti-GFP group. There was no obvious difference of TUNEL-positive cells under ISO (1umol/L) stimulating between LV-anti-GFP and lenti-GFP group.
(TIF)

S1 Table Gene symbol, name and primer sequences. (DOC)
