# Supplementary figures and images for: Overexpression of microRNA-99a Attenuates Cardiac Hypertrophy
Source: PLoS One. 2016 Feb 25;11(2):e0148480. doi: 10.1371/journal.pone.0148480 (PMC4767297; doi:10.1371/journal.pone.0148480)

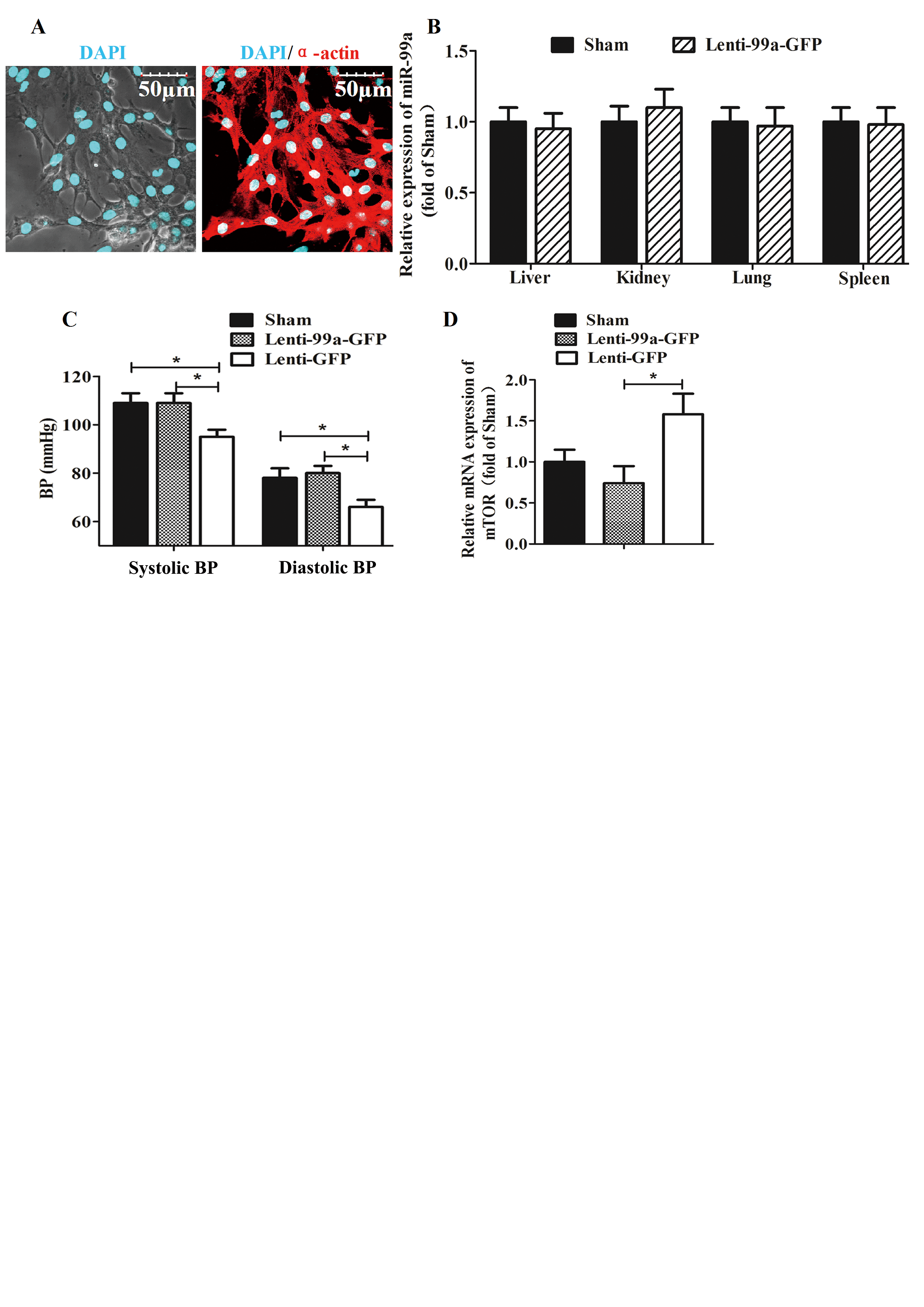

Supplement: S1 Fig — A. Purity and lentiviral infection of NMVMs. The purity of α- actin + cells was ≥95% in culture cell population. B. One week after lentivirus (lenti-GFP-99a) intramyocardially infection, there was no difference of miR-99a expression in kidney, lung, liver and spleen. C. We observed that miR-99a overexpression in hearts of mice did alter the BP (systolic BP: 109 ± 3 mmHg and 95 ± 2 mmHg, diastolic BP: 80 ± 2 mmHg and 66 ± 2 mmHg, lenti-99a-GFP group verse lenti-GFP group, *, p<0.05). However, systemic blood pressure of sham group and lenti-99a-GFP group were not different. D. Seven weeks after infection, we observed a 50%-fold decrease in mRNA transcriptional level of mTOR in lenti-99a-GFP group compared to lenti-GFP group (*, p<0.05). (TIF) [file pone.0148480.s001.tif]

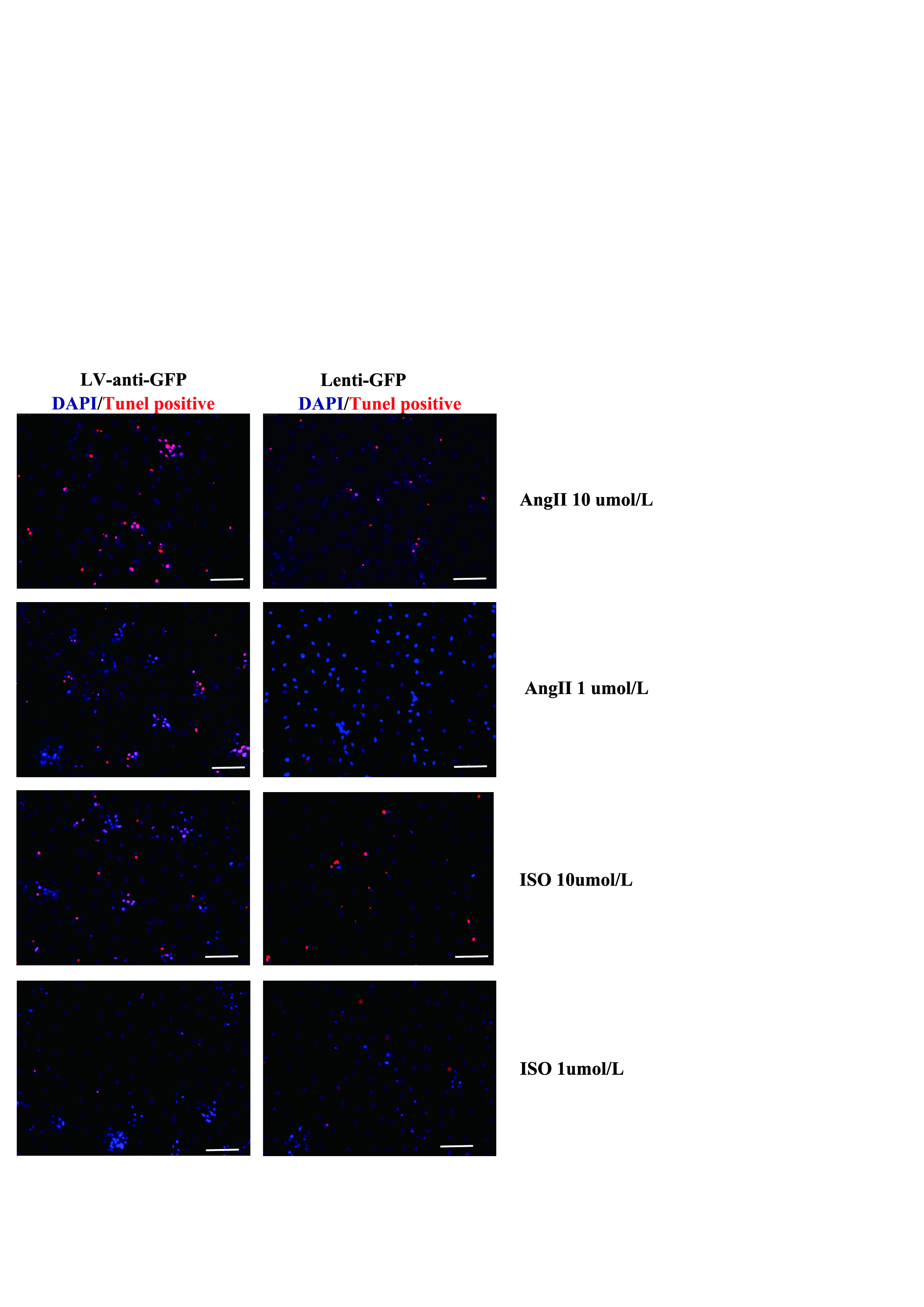

Supplement: S2 Fig — Cardiomyocytes were infected with LV-anti-GFP or Lenti-GFP for 72 hours, and then treated with Ang II (1/10umol/L) or ISO (1/10umo/L) for 12 hours. We found that cardiomyocytes suffered from Ang II (1umol/L and 10 umol/L) and ISO (10umol/L) showed more TUNEL-positive cells in LV-anti-GFP group than lenti-GFP group. There was no obvious difference of TUNEL-positive cells under ISO (1umol/L) stimulating between LV-anti-GFP and lenti-GFP group. (TIF) [file pone.0148480.s002.tif]
