# Supplementary material for: Overexpression of microRNA-99a Attenuates Cardiac Hypertrophy
Source: PLoS One. 2016 Feb 25;11(2):e0148480. doi: 10.1371/journal.pone.0148480 (PMC4767297; doi:10.1371/journal.pone.0148480)
Supplement: S1 Table — (DOC) [file pone.0148480.s003.doc]

| Primer | Gene name | Forward primer 5' to 3' | Reverse primer 5' to 3' |
| --- | --- | --- | --- |
| β-actin | Beta actin | GTGGGCCGCTCTAGGCACCAA | CTCTTTGATGTCACGCACGATTTC |
| β-MHC | Beta-2 microglobulin | TGAGCGGCGCATCAAGGAGC | CTTGGCACCAATGTCCCGGC |
| SERCA 2a | Sarcoplasmic reticulum Ca2+ ATPase | GCGTGCAAACGCCTGCAACT | CCAGGCACCGTAGCGTGTCG |
| mTOR | Mammalian target of rapamycin | AAGGCCTGATGGGATTTGG | TGTCAAGTACACGGGGCAAG |
| PPARα | Peroxisome proliferator activated receptor α | CGGAGTGCAGCCTCAGCCAAGTTGA | ATGTTGGATGGATGTGGCCAGGCA |
| HIF-1α | Hypoxia-inducible factor-1α | AGACAACGCGGGCACCGATTC | GTGGGGAAGTGGCAACTGATGAGC |
| GLUT1 | Glyceraldehyde phosphate dehydrogenase | ATGGCAGGCTGTGCTGTGCTC | GCCGCACAGTTGCTCCACAT |
| HK2 | Hexokinase 2 | CAGCTCTGTGGCGCAGGCAT | TATCTCTGCCCGGCCTCCCG |
| BNP | Brain natriuretic peptide | GCATGCCCAACCCACTTTAC | GAGAGACAGGGCAATGTCAC |
| ACTA1 | α-skeletal actin | CCCAGGGCCAGAGTCAGAGCAGCAG | GCTCTGGGCCTCATCACCCACG |

S1 Table 1. Gene symbol, name and primer sequences.
